# Ovarian Splenosis: A Case Report

**DOI:** 10.1155/2010/472162

**Published:** 2010-06-13

**Authors:** H. Talati, Jasim Radhi

**Affiliations:** Department of Pathology, McMaster University, 1200 Main Street West, Hamilton, ON, Canada L8N 3V7

## Abstract

Splenosis is auto transplantation of splenic tissue following traumatic rupture of the spleen. In females it can mimic endometriosis when symptomatic. Asymptomatic splenosis is common than previously suspected and it can also involve ovary. In a patient with a history of splenectomy, splenosis can act and provide the function of the spleen and thus should not be routinely excised. We report a case of an asymptomatic, incidental ovarian splenosis of left ovary accompanying multiple pelvic and serosal splenotic nodules. To our best knowledge, total three cases of ovarian splenosis have been reported previously including two cases of ovarian splenosis accompanying pelvic and serosal splenotic nodules and one case of solitary ovarian splenosis.

## 1. Introduction

Splenosis is a term initially used by Buchbinder and Lipkoff in 1939 to describe the heterotopic transplantation of splenic tissue as a consequence of splenic trauma or surgery [1]. Splenic tissue can get implanted in the form of encapsulated nodules. It can occur on the peritoneal surface, pelvis, abdominal wall, pleural cavity, lung parenchyma, and rarely in the brain. Splenosis is most often encountered in the peritoneal cavity after splenic rupture. Intrathoracic and subcutaneous splenosis occurs when trauma causes a path of entry for implantation of splenic tissue. However, in cerebral splenosis a haematogenous spread of splenic tissue has to be assumed [2]. 

Gynecological patients present with pelvic nodules mimicking endometriosis, presenting as pelvic pain or discovered incidentally, and are asymptomatic as in most cases [2]. Splenosis may create false impression of ovarian neoplasm during ultrasound imaging [3]. As splenosis is usually asymptomatic and may partially compensate for the asplenic state, it is recommended that asymptomatic splenosis should not be resected when encountered during surgery [4].

## 2. Case Presentation

A 53-year-old Caucasian woman presented in the emergency department with acute right lower quadrant abdominal pain. Physical examination revealed right lower quadrant tenderness with guarding and rigidity. Laboratory investigations showed elevated white blood cell count with neutrophilia. Urine examination was negative. Abdominal ultrasonography revealed right adnexal mass. Patient denied history of any genitourinary tract infection and pelvic inflammatory disease. Patient had undergone a splenectomy at the age of 18 years, after splenic trauma in a road traffic accident, and had four cesarean sections after the age of 25 years with no intra- /postoperative complications.

She underwent bilateral salpingoopherectomy for right adnexal mass. Intraoperative findings included multiple adhesions in upper abdomen from previous surgery along with hydrosalpinx on right side. Colon was densely attached to left pelvic side wall with adhesions to right ovary. Multiple cysts were identified in both ovaries. Postoperative course was uneventful.

**Figure 1:**
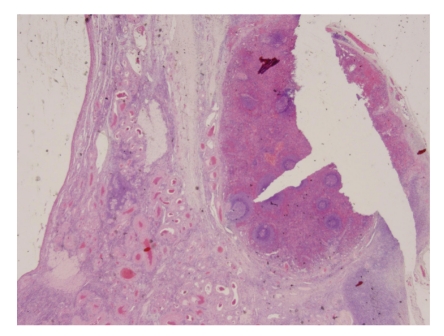
Left ovarian tissue with serosal splenic nodule.

**Figure 2:**
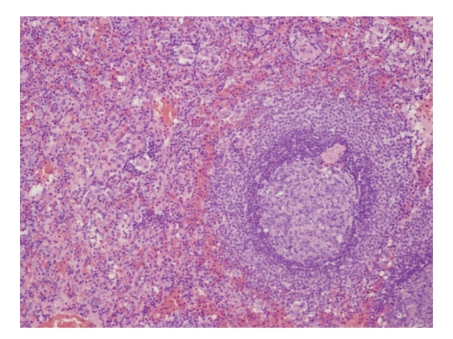
Splenic nodule showing white pulp composed of lymphoid aggregates with eccentric arterioles surrounded by red pulp.

Microscopic examination of right fallopian tube showed hydrosalpinx with multiple serosal adhesions. Right and left ovaries showed presence of multiple follicular cysts and inclusion cyst, respectively. Left ovary also showed incidental well-defined splenotic nodule on the serosa, 5.0 mm in maximum dimension, with features resembling normal spleen (Figure 1). White pulp composed of lymphoid aggregates with eccentric arterioles was surrounded by highly vascular red pulp composed of broad anastomosing venous sinuses (Figure 2). A true capsule was not identified but short trabeculae are noted in the parenchyma. 

Follow-up abdominal imaging postoperatively following pathology diagnosis revealed presence of multiple round lesions in left upper quadrant and middle abdomen including greater curvature of stomach in keeping with splenosis.

## 3. Discussion

Only two cases of ovarian splenosis accompanied by multiple pelvic and serosal nodules clinically mimicking endometriosis have been described previously [4, 5]. 

The differential diagnoses to be considered in this case are accessory spleen and splenogonadal fusion.

Histologically, in splenosis the splenic tissue may be poorly developed architecturally or, as in this case, indistinguishable from the parent organ. Although many authors state that various features can differentiate splenosis from accessory spleen histologically, Carr and Turk [6] report two cases in which, as in our case, histology was unhelpful because there were no distinguishing features between the two. 

Accessory spleen occurs in about 10% of individuals and can be solitary or multiple with histological and functional features similar to those of the normal spleen. This occurs as a result of the normal multifocal development of spleen and subsequent failure of fusion of one or more contributory foci. The most common site is the splenic hilum. Less frequently it occurs in the tail of the pancreas and has been reported in the liver and retroperitoneum. 

Splenogonadal fusion is the result of fusion of the splenic and gonadal anlage during embryonic development. Most reported cases are in males, which may not necessarily reflect a true difference in incidence but may be, as noted by Watson [7], the result of the fact that the situation of the male gonads makes them more accessible to examination. In addition, except for one case, the fusion involved the left gonad [8]. Splenogonadal fusion occurs in two forms. One is continuous, in which a fibrous band connects the gonadal and splenic structures. The other is discontinuous, in which discrete masses of splenic tissue are found fused to the gonadal structures. The first form is associated with congenital malformations, including limb defects and micrognathia. Meneses and Ostrowski [9] described the first reported case of discontinuous splenogonadal fusion in a female, which occurred as intraovarian splenic tissue in the left ovary. Several criteria help in differentiating splenosis from splenogonadal fusion. In splenosis, splenic tissue is found diffusely throughout the peritoneal cavity and peritoneal surfaces as opposed to specific location in splenic-gonadal fusion. The splenic tissue is variously shaped in splenosis with no hilum or defined capsule as opposed to splenic-gonadal fusion which architecturally simulates normal spleen. As splenosis is usually asymptomatic and may partially compensate for the asplenic state, it is recommended that asymptomatic splenosis should not be resected when encountered during surgery [4].

Our case represents a case of asymptomatic ovarian splenosis found incidentally in left ovary accompanying multiple pelvic and abdominal splenotic nodules.
